# Effects of chemokines on proliferation and apoptosis of human mesangial cells

**DOI:** 10.1186/1471-2369-5-8

**Published:** 2004-07-20

**Authors:** Markus Wörnle, Holger Schmid, Monika Merkle, Bernhard Banas

**Affiliations:** 1 Medical Policlinic, Ludwig-Maximilians-University, Munich, Germany

**Keywords:** Mesangial cell, proliferation, apoptosis, chemokine, chemokine receptor

## Abstract

**Background:**

Proliferation and apoptosis of mesangial cells (MC) are important mechanisms during nephrogenesis, for the maintenance of glomerular homeostasis as well as in renal disease and glomerular regeneration. Expression of chemokines and chemokine receptors by intrinsic renal cells, e.g. SLC/CCL21 on podocytes and CCR7 on MC is suggested to play a pivotal role during these processes. Therefore the effect of selected chemokines on MC proliferation and apoptosis was studied.

**Methods:**

Proliferation assays, cell death assays including cell cycle analysis, hoechst stain and measurement of caspase-3 activity were performed.

**Results:**

A dose-dependent, mesangioproliferative effect of the chemokine SLC/CCL21, which is constitutively expressed on human podocytes was seen via activation of the chemokine receptor CCR7, which is constitutively expressed on MC. In addition, in cultured MC SLC/CCL21 had a protective effect on cell survival in Fas-mediated apoptosis. The CXCR3 ligands IP-10/CXCL10 and Mig/CXCL9 revealed a proproliferative effect but did not influence apoptosis of MC. Both the CCR1 ligand RANTES/CCL5 and the amino-terminally modified RANTES analogue Met-RANTES which blocks CCR1 signalling had no effect on proliferation and apoptosis.

**Conclusions:**

The different effects of chemokines and their respective receptors on proliferation and apoptosis of MC suggest highly regulated, novel biological functions of chemokine/chemokine receptor pairs in processes involved in renal inflammation, regeneration and glomerular homeostasis.

## Background

Originally chemokines (*chemo*tactic cyto*kines*) were described as key mediators for the selective migration of leukocytes into sites of tissue injury [1]. Later on chemokines and chemokine receptors have also been described as important mediators in noninflammatory processes, including normal cellular trafficking, hematopoesis, angiogenesis, organ development, tissue remodelling, and tumor metastasis [2-4]. To date more than 40 different human chemokines are characterized. The chemokine superfamily is separated into the C, CC, CXC, and CX3C subfamilies (Where X represents any intervening amino acid residue between the first two cysteines in the amino acid sequence) [5,6]. Chemokines mediate their biological activity by ligation and interaction with seven-transmembrane-spanning G protein-coupled receptors (i.e. C, CC, CXC, and CX3C receptors) [7].

In the kidney expression of chemokines and chemokine receptors are important for the initiation and regulation of inflammatory glomerular diseases [8]. Expression of the chemokines monocyte chemoattractant protein-1 (MCP-1/CCL2), regulated upon activation, normal T cell expressed and secreted (RANTES/CCL5), interleukin-8 (IL-8), and interferon-γ (IFN-γ)-inducible protein of 10 kD (IP-10/CXCL10) by human mesangial cells (MC) was shown by our group [9] and others [1,10]. Inducible expression of the chemokine receptor CCR1 by human MC was previously described [9]. The expression of the chemokine receptor CXCR3 on human MC was published by Romagnani and colleagues [11]. A high level of expression of this receptor by MC was seen by immunohistochemistry in kidney biopsies from patients with IgA nephropathy, membranoproliferative glomerulonephritis or rapidly progressive glomerulonephritis. Recently our group showed that SLC/CCL21 is constitutively expressed by glomerular podocytes and CCR7 constitutively expressed by MC [12].

In the kidney the well-regulated relationship among resident cell proliferation and apoptosis is important for the development of the sophisticated glomerular architecture during ontogenesis as well as maintaining normal function of adult human glomeruli. Dysfunction of the balance between glomerular cell proliferation and apoptosis after leukocyte infiltration has been discussed for many inflammatory kidney diseases [13].

The finding of expression of chemokine receptors and their respective ligands by intrinsic renal cells not only under inflammatory conditions led to the hypothesis of an involvement of these receptors in glomerular homeostasis. Therefore the influence of chemokines on mesangial cell growth was investigated. We describe the different effects of the chemokines SLC/CCL21, IP-10/CXCL10, Mig/CXCL9, RANTES/CCL5 and the amino-terminally modified RANTES/CCL5 analogue Met-RANTES on mesangial cell proliferation and apoptosis, suggesting novel functions of chemokine/chemokine receptor pairs on local immunomodulation, glomerular regeneration and homeostasis.

## Methods

### Cell culture conditions for human mesangial cells

Immortalized human mesangial cells (MC) were grown as described previously [9]. This MC line was characterized for antigenic markers typically expressed by MC *in vivo *and *in vitro *and showed no dedifferentiation within approximately 100 passages during a 36 months cultivation period. For all experiments cells in passages 51 to 65 were used. Different preparations of primary human MC served as controls and were cultured as previously described [14].

### Proliferation assay

To assess proliferation we performed a MTT (3-(4,5-dimethylthiazol-2-yl)-2,5-diphenyl-tetrazolium bromide, Sigma, Germany) assay [15]. Aliquots of 20 × 10^3 ^cells in 100 μl medium were cultured in 96-well microtitre plates for 24 hours under standard conditions to yield firmly attached and stably growing cells. Subsequently the medium was changed to 100 μl of medium containing test substances and the cells were incubated from 24 to 72 hours. After discarding the supernatants 50 μl of a 1 mg/ml solution of MTT were added. The cells were incubated for 3 hours at 37°C and then formazan crystals were dissolved by addition of 50 μl isopropanol. Absorbance was measured at 550 nm against 630 nm reference using a DYNATECH (Germany) MR7000 ELISA reader. For each experiment at least 6 wells were analyzed per experimental condition and time point.

### Cell death assays

Apoptosis of MC was induced by Fas/CD 95 ligation as previously described [16]. Fas/CD 95 surface expression was induced by starvation of the cells in serum-free medium and pre-stimulation with IFN-γ (70 ng/ml) for 48 h. For analysis of chemokine effects, cells were pretreated with SLC/CCL21 250 ng/ml, IP-10/CXCL10 100 ng/ml, Mig/CXCL9 100 ng/ml, RANTES/CCL5 100 ng/ml, and Met-RANTES 100 ng/ml prior to adding the activating anti-human Fas antibody (400 ng/ml, incubation over 18 hours) (Biomol, Germany). To induce expression of the chemokine receptor CCR1 in a different experiment MC were preincubated with a combination of IFN-γ (20 ng/ml), TNF-α (25 ng/ml) and IL-1β (10 ng/ml) for 24 h before starvation of the cells in serum-free medium and stimulation with IFN-γ (70 ng/ml), TNF-α (25 ng/ml) and IL-1β (10 ng/ml) for 48 h. Apoptosis was studied with several assays: Flow cytometric cell cycle analysis was performed using propidium iodide staining as described previously [17]. For visualization of chromatin fragmentation, MC were seeded on chamber slides (NUNC, Germany). After treatment with test substances cells were fixed with ethanol and stained with the nuclear dye Hoechst 33258 (Hoechst, Germany) 5 μmol/ml. The percentage of apoptotic cells was determined by immunofluorescence microscopy, counting nuclei with condensed and fragmented chromatin. Three different experiments were performed, at least 300 cells were analyzed per condition. Counting was performed in a blinded manner by two investigators. For measurement of caspase-3 activity [18] a commercial assay (R&D systems, Germany) was used according to the manufacturer's specifications. After induction of apoptosis as described above, MC were lysed and caspase-3 specific proteolytic activity was quantitated spectrophotometrically. Three experiments were done analyzing duplicates for any experimental condition.

### Statistical analysis

Values are provided as mean ± SEM. Statistical analysis was performed by unpaired *t *test. Significant differences are indicated for p values < 0.05 (*) or < 0.01 (**), respectively.

## Results

### Effects of SLC/CCL21, IP-10/CXCL10, Mig/CXCL9, RANTES/CCL5 and Met-RANTES on proliferation of human mesangial cells

#### SLC/CCL21 induces proliferation of human MC

**Figure 1:**
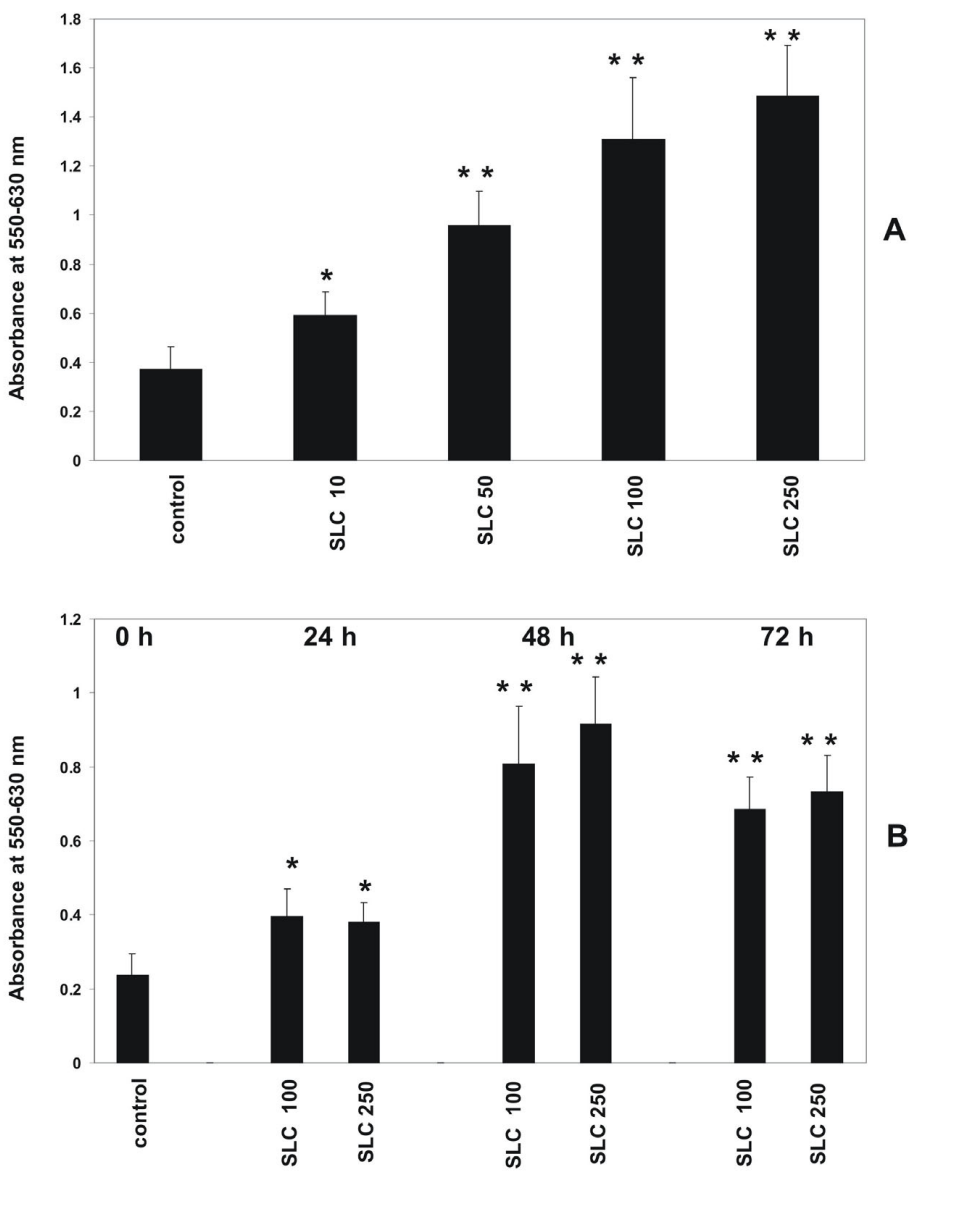
Dose- and time- dependent effect of SLC/CCL21 on proliferation of human MC. (A) Incubation of human MC with various concentrations of SLC/CCL21 (10 ng/ml, 50 ng/ml, 100 ng/ml, 250 ng/ml) induces proliferation of MC in a dose-dependent manner. (B) Time-course stimulation of MC with SLC/CCL21 for various time intervals (24 h, 48 h, 72 h). Cell proliferation was analyzed with the MTT assay as described in Methods. Cells growing under standard conditions served as control. Changes in proliferative activity are given as relative values to the respective controls. Each bar represents a mean ± SEM of 7 parallel incubations for each condition. Statistically significant differences to the control are depicted with * = p < 0.05 and ** = p < 0.01, resp. Comparable results were obtained in three series of independent experiments.

To demonstrate that activation of CCR7 has an influence on the proliferative activity of MC MTT assays were performed as described in Methods. The CCR7 ligand SLC/CCL21 led to a concentration dependent increase of proliferation of human MC in a range from 10 to 250 ng/ml after 24 hours of stimulation (Figure 1A). In a time course experiment from 24 to 72 hours the maximum increase of proliferation was seen after incubation with SLC/CCL21 for 48 hours (Figure 1B).

#### IP-10/CXCL10 and Mig/CXCL9 induce proliferation of human MC

**Figure 2:**
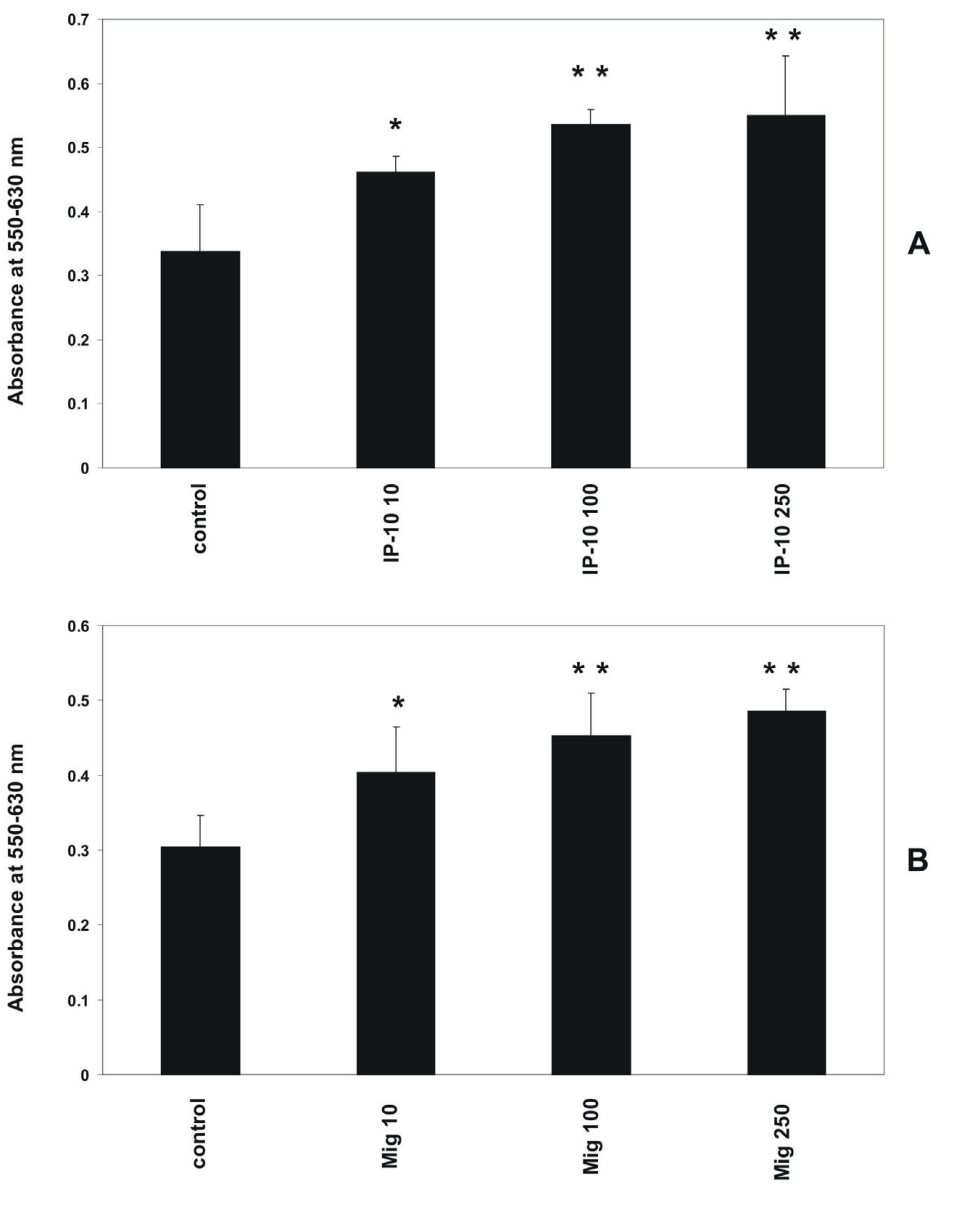
Dose-dependent effect of IP-10/CXCL10 and Mig/CXCL9 on proliferation of human MCIncubation of human MC for 24 hours with various concentrations of IP-10/CXCL10 and Mig/CXCL9 (10 ng/ml, 100 ng/ml, 250 ng/ml) induces proliferation of MC in a dose-dependent manner. Cell proliferation was analyzed with the MTT assay as described in Methods. Cells growing under standard conditions served as control. Changes in proliferative activity are given as relative values to the respective controls. Each bar represents a mean ± SEM of 5 parallel incubations for each condition. Statistically significant differences to the control are depicted with * = p < 0.05 and ** = p < 0.01, resp. Comparable results were obtained in three series of independent experiments.

CXCR3 activation had an influence on the proliferative activity of MC. As shown in Figure 2, stimulation with the CXCR3 ligands IP-10/CXCL10 and Mig/CXCL9 had an increasing effect on the proliferation of human MC in concentrations 10 to 250 ng/ml (Figure 2). Incubation period was 24 hours.

#### RANTES/CCL5 and Met-RANTES have no effect on proliferation of human MC

**Figure 3:**
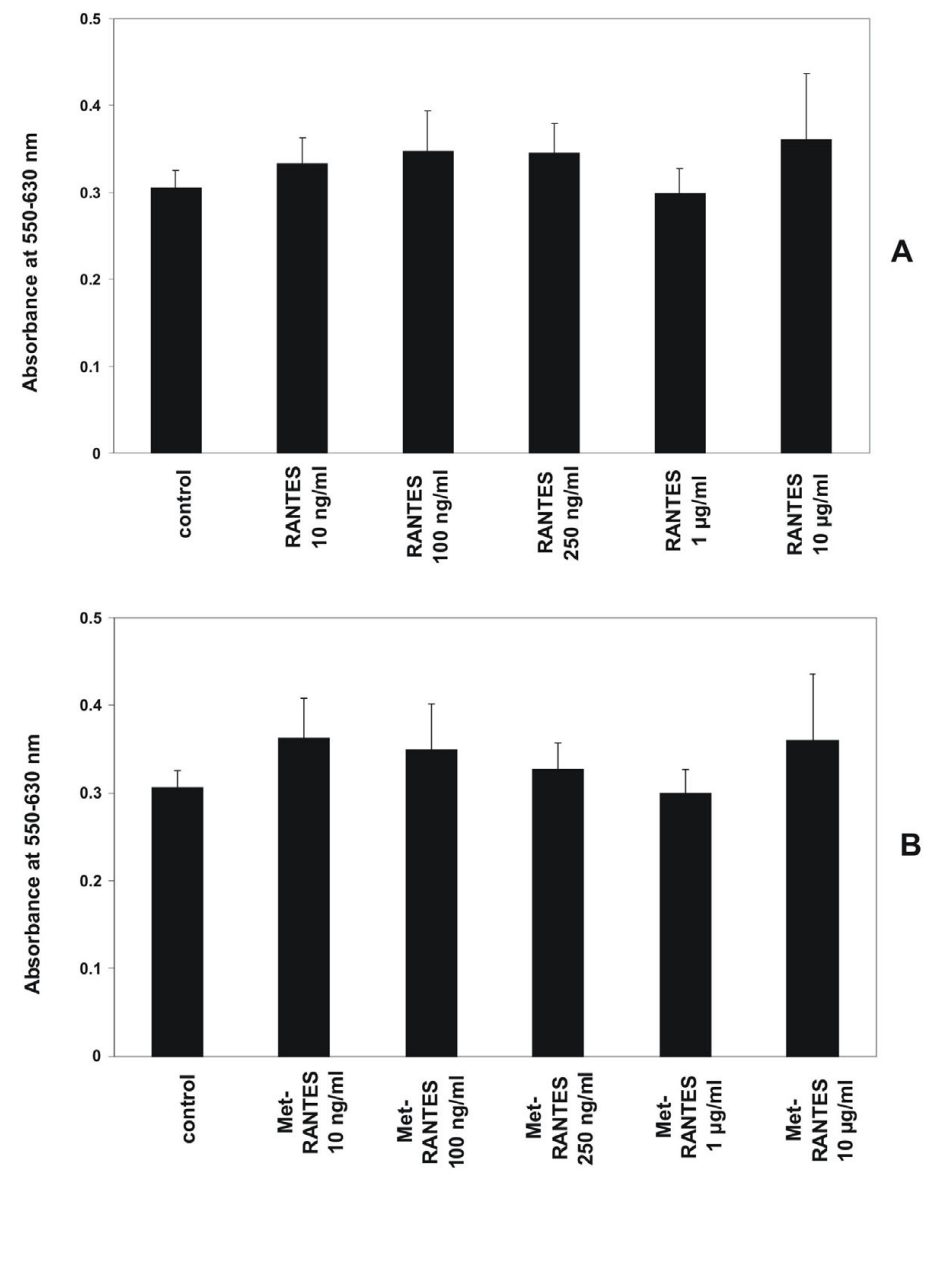
Effect of RANTES/CCL5 and Met-RANTES on proliferation of human MCIncubation of human MC for 24 hours with various concentrations of RANTES/CCL5 (10 ng/ml, 100 ng/ml, 250 ng/ml, 1 μg/ml, 10 μg/ml) and Met-RANTES (10 ng/ml, 100 ng/ml, 250 ng/ml, 1 μg/ml, 10 μg/ml) has no effect on proliferation of MC under standard conditions. To induce expression of chemokine receptor CCR1 MC were prestimulated with IFN-γ (20 ng/ml), TNF-α (25 ng/ml) and IL-1β (10 ng/ml) for 24 hours prior to adding test substances. Cell proliferation was analyzed with the MTT assay as described in Methods. Cells growing under standard conditions served as control. Changes in proliferative activity are given as relative values to the respective controls. Each bar represents a mean ± SEM of 7 parallel incubations for each condition. Comparable results were obtained in three series of independent experiments.

Incubation of human MC with various concentrations of the CCR1 ligands RANTES/CCL5 (10 ng/ml, 50 ng/ml, 100 ng/ml, 250 ng/ml) and Met-RANTES (10 ng/ml, 50 ng/ml, 100 ng/ml, 250 ng/ml) had no effect on the proliferation of MC at an incubation period of 24 hours under standard conditions (Figure 3). To induce chemokine receptor CCR1 expression cells were pretreated with a combination of IFN-γ (20 ng/ml), TNF-α (25 ng/ml) and IL-1β (10 ng/ml) for 24 hours prior to adding test substances. Stimulation with RANTES/CCL5 or Met-RANTES without pretreatment with cytokines has no effect on proliferation (data not shown).

### Effects of SLC/CCL21, IP-10/CXCL10, Mig/CXCL9, RANTES/CCL5 and Met-RANTES on apoptosis of human mesangial cells

#### SLC/CCL21 has an anti-apoptotic effect on Fas/CD95-induced apoptosis

**Figure 4:**
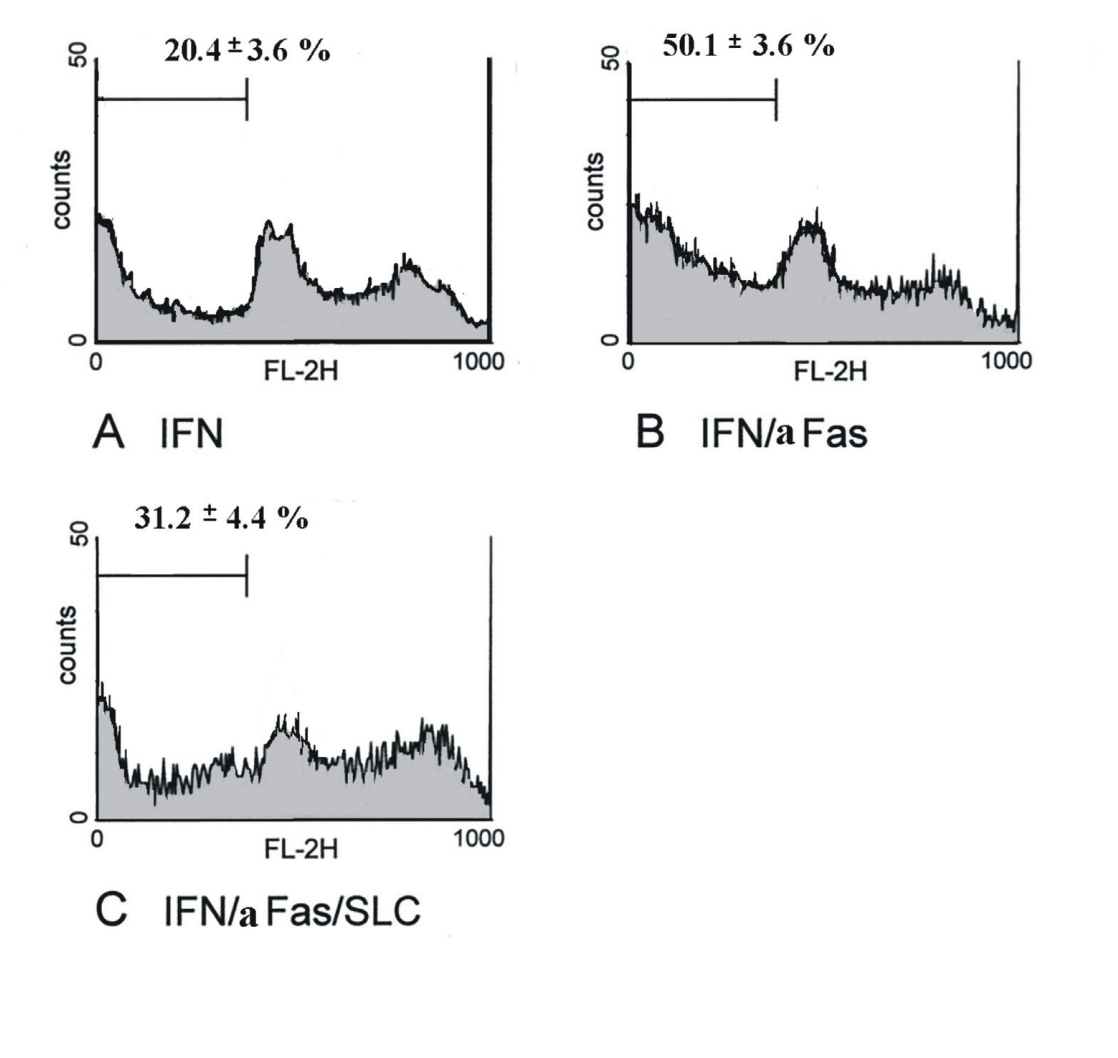
Effect of SLC/CCL21 on Fas-induced cell death of human MC in cell cycle analysis by flow cytometry. Percentage of apoptotic cells was analyzed in cell cycle analysis by flow cytometry after staining with propidium iodide. Histograms represent cell counts (y-axis) versus DNA content (x-axis) with the percentage of apoptotic cells containing sub-G1 DNA indicated. Figure 4A shows the cell cycle analysis of MC after serum starvation and stimulation with IFN-γ for 48 hours. Figure 4B: Apoptosis was induced subsequently by Fas ligation. Figure 4C: Effect of preincubation with SLC/CCL21 250 ng/ml on Fas-induced apoptosis of MC. The profiles shown are representative for four independent experiments.

**Figure 5:**
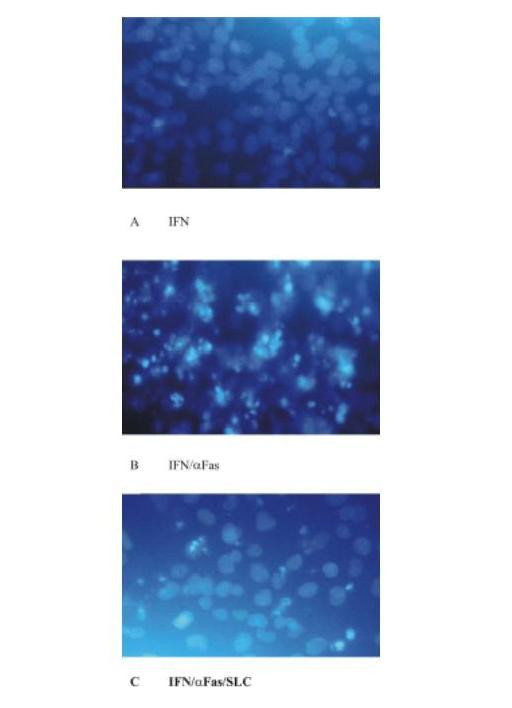
Effect of SLC/CCL21 on Fas-induced cell death of human MC in Hoechst stain on a fluorescent microscopic analysis of MC (A) MC stimulated with IFN-γ. (B) MC stimulated wit IFN-γ and Fas ligation. (C) Significantly reduced number of apoptotic cells when MC were prestimulated with SLC/CCL21 prior to induction of cell death.

**Figure 6:**
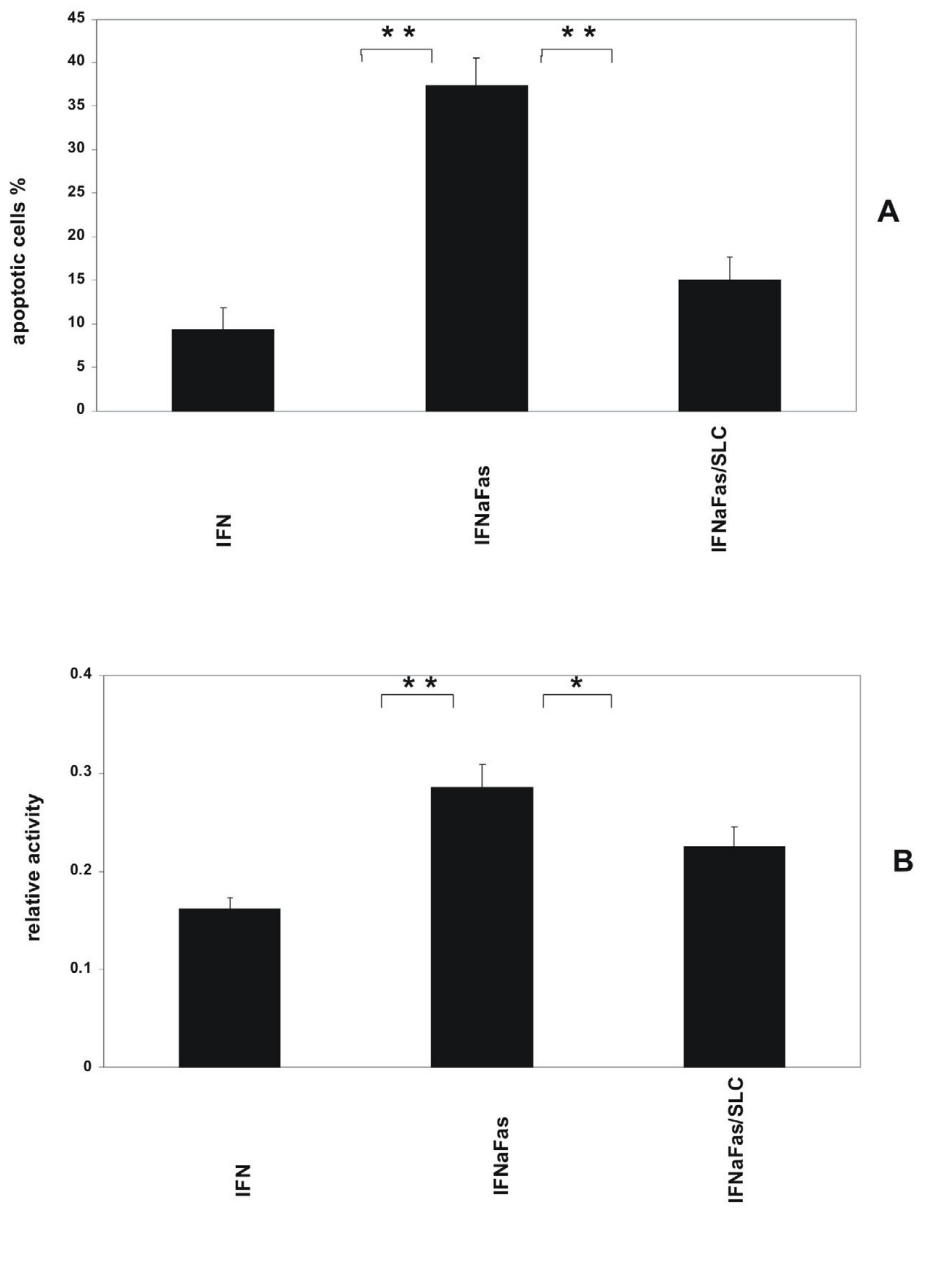
Effect of SLC/CCL21 on Fas-induced cell death of human MC in cell count and caspase-3 assay. (A) The percentage of apoptotic MC was determined after visualisation of fragmented chromatin with Hoechst dye. Apoptotic nuclei were analyzed microscopically in three different sets of experiments counting at least 300 cells per condition. (B) Caspase-3 activity was quantitated spectrophotometrically in MC lysates. Data are from three independent sets of experiments, each performed in duplicate. Statistically significant differences are depicted: * = p < 0.05 and ** = p < 0.01, resp.

The effect of CCR7 activation for MC survival in Fas/CD95-induced cell death was studied with three different methods. Flow cytometric cell cycle analysis using propidium iodide staining showed 8.3 ± 1.1% apoptotic cells under normal conditions. After serum-starvation and incubation with IFN-γ for 48 hours human MC express Fas/CD95 on surface (data not shown). Under these conditions the population with a sub-G1 DNA content was 20.4 ± 3.6% (Figure 4A). Serum-starvation and stimulation with IFN-γ showed no difference in the number of apoptotic MC compared to serum-starvation without stimulation with IFN-γ (data not shown). Subsequent stimulation with an activating anti-Fas antibody increased the amount of MC with a sub-G1 DNA content to 50.1 ± 3.6% consistent with a marked increases in apoptosis (Figure 4B). When MC were prestimulated with SLC/CCL21 prior to induction of cell death the percentage of apoptotic cells was reduced markedly to 31.2 ± 4.4% (Figure 4C). The results are from four independent experimental series. The data are shown in Table 1. Staining with Hoechst visualizes cells with fragmented chromatin. Figure 5 shows a fluorescent microscopic analysis of MC. Figure 5A represents cells stimulated with IFN-γ and Figure 5B cells stimulated with IFN-γ and Fas ligation. Figure 5C shows a significantly reduced number of apoptotic cells when MC were prestimulated with SLC/CCL21 prior to induction of cell death. Apoptotic nuclei were analyzed microscopically in three different sets of experiments counting at least 300 cells per condition. After serum starvation and IFN-γ stimulation 9.4 ± 2.5% of the cells were found to be apoptotic. Subsequent Fas ligation induced apoptosis in 37.3 ± 3.2% of human MC. Prestimulation with SLC/CCL21 reduced Fas-induced cell death effectively to 15.0 ± 2.6% (Figure 6A). Induction of cell death of MC by Fas ligation increased Caspase-3 activity 2.8-fold compared with control conditions. Coincubation with SLC/CCL21 reduced caspase-3 activity significantly (Figure 6B)

**Table 1:** Effect of SLC/CCL21, IP-10/CXCL10, Mig/CXCL9, RANTES/CCL5 and Met-RANTES on Fas-induced cell death of human mesangial cells.

	**IFN**	**IFN/aFas**	**IFN/aFas/ SLC**	**IFN**	**IFN/aFas**	**IFN/aFas/IP-10**	**IFN/aFas/Mig**	**IFN**	**IFN/aFas**	**IFN/aFas/RANTES**	**IFN/aFas/Met-RANTES**
	15.8	47.2	24.7	11.1	36.7	21.1	25.8	18.3	40.9	31.6	35.5
	22.4	53.1	33.7	16.3	33.1	25.3	28.2	19.3	34.1	40.7	41.8
	23.9	53.4	34.1	20.2	29.9	28.7	28.9	24.8	46.2	39.6	35.5
	19.6	46.8	32.4	13.5	25.9	31.1	36.3	16.2	37.9	34.2	42.6
**Mean**	**20.4**	**50.1**	**31.2**	**15.3**	**31.4**	**26.6**	**29.8**	**19.7**	**39.8**	**36.5**	**38.1**
SEM	3.6	3.6	4.4	3.9	4.6	4.3	4.5	3.7	5.1	4.3	4.9

Data of four separate FACS analyses, respectively. Values are means ± SEM.

#### IP-10/CXCL10 and Mig/CXCL9 have no effect on Fas/CD95-induced cell death of human MC

**Figure 7:**
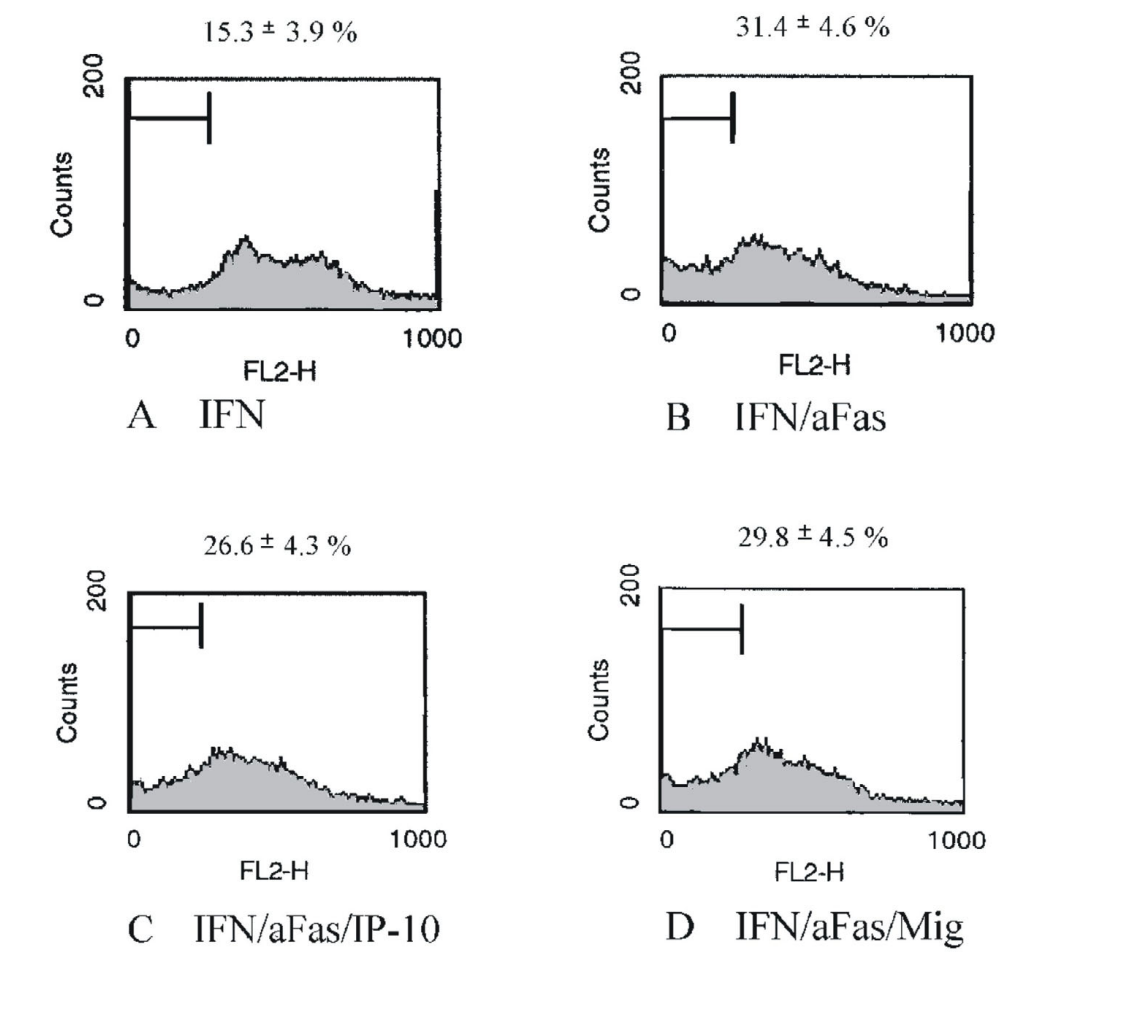
Effect of IP-10/CXCL10 and Mig/CXCL9 on Fas-induced cell death of human MC in cell cycle analysis by flow cytometry. Percentage of apoptotic cells was analyzed in cell cycle analysis by flow cytometry after staining with propidium iodide. Histograms represent cell counts (y-axis) versus DNA content (x-axis) with the percentage of apoptotic cells containing sub-G1 DNA indicated. Cell cycle analysis of MC was performed after serum starvation and stimulation with IFN-γ for 48 hours. (A) Cell cycle analysis of MC after serum starvation and stimulation with IFN-γ for 48 hours. (B) Apoptosis was induced subsequently by Fas ligation. (C, D) Preincubation with IP-10/CXCL10 and Mig/CXCL9 has no effect on Fas-induced apoptosis of MC. The profiles shown are representative for four independent experiments.

To investigate the effect of the CXCR3 ligands IP-10/CXCL10 and Mig/CXCL9 on Fas-induced cell death MC were grown without serum and stimulated with IFN-γ. Induction of cell death by an activating anti-Fas-antibody led to significantly increased number of apoptotic cells. Coincubation with IP-10/CXCL10 or Mig/CXCL9 had no effect on Fas-induced apoptosis of MC (Figure 7). The data of four separate experiments are shown in Table 1.

#### RANTES/CCL5 and Met-RANTES have no effect on Fas/CD95-induced cell death of human MC

**Figure 8:**
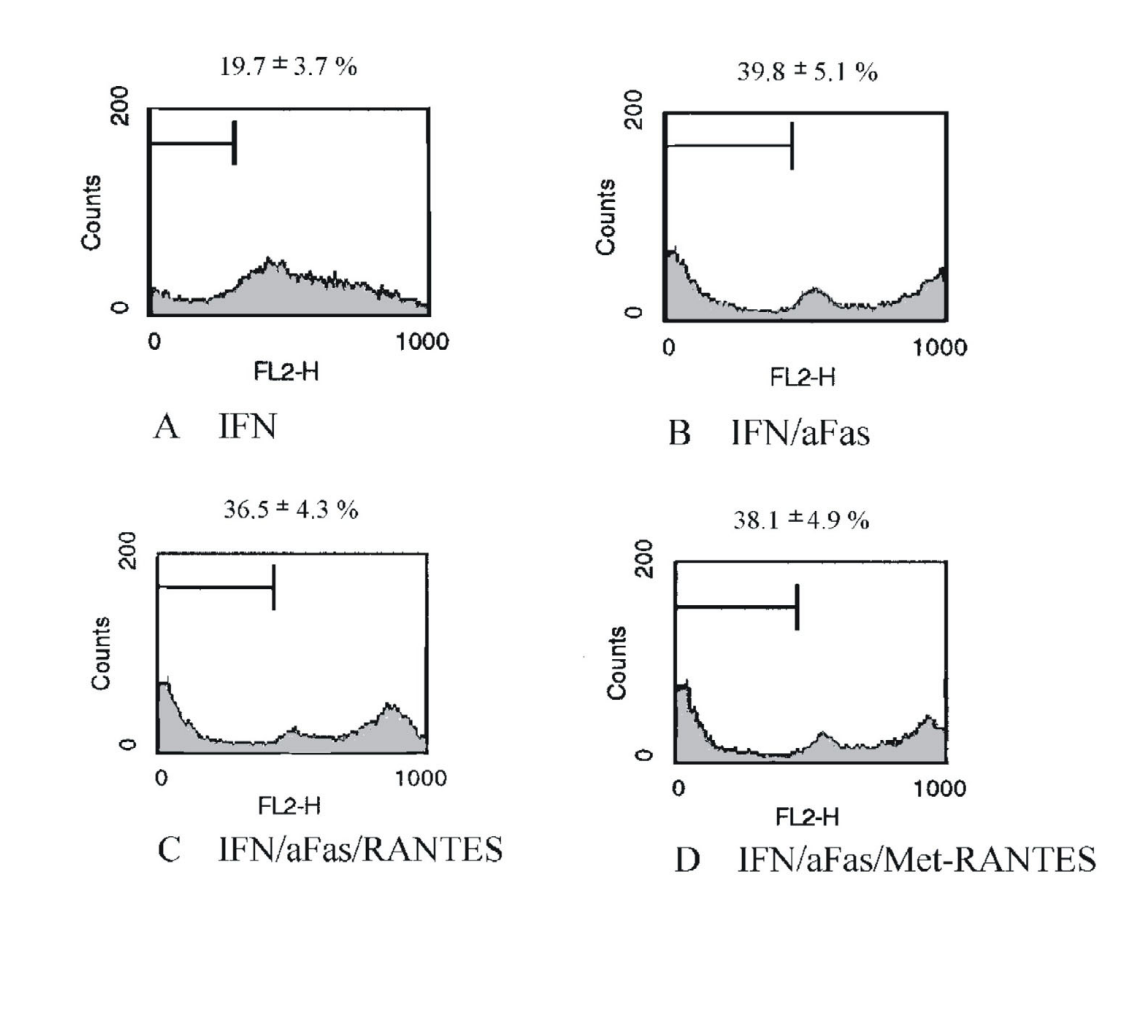
Effect of RANTES/CCL5 and Met-RANTES on Fas-induced cell death of human MC in cell cycle analysis by flow cytometry. Percentage of apoptotic cells was analyzed in cell cycle analysis by flow cytometry after staining with propidium iodide. Histograms represent cell counts (y-axis) versus DNA content (x-axis) with the percentage of apoptotic cells containing sub-G1 DNA indicated. (A) Cell cycle analysis of MC was performed after serum starvation and stimulation with IFN-γ for 48 hours. (B) Apoptosis was induced subsequently by Fas ligation. Prior to serum starvation the expression of chemokine receptor CCR1 was induced by pretreatment with a combination of IFN-γ (20 ng/ml), TNF-α (25 ng/ml) and IL-1β (10 ng/ml) for 24 hours. (C,D) RANTES/CCL5 250 ng/ml and Met-RANTES 250 ng/ml have no effect on Fas-induced cell death of MC.

To induce expression of chemokine receptor CCR1 cells were pretreated with a combination of IFN-γ (20 ng/ml), TNF-α (25 ng/ml) and IL-1β (10 ng/ml) for 24 hours, followed by serum-starvation and incubation with IFN-γ for 48 hours (Figure 8A). Cell death was induced again by stimulation with an activating anti-Fas antibody (Figure 8B). Stimulation with RANTES/CCL5 (250 ng/ml) (Figure 8C) or Met-RANTES (250 ng/ml) (Figure 8D) prior to induction of cell death was ineffective in maintaining cell survival. The results are from four independent experimental series (See Table 1). The percentage of apoptotic cells varies between the experiments due to biological variance.

## Discussion

Proliferation and apoptosis play a pivotal role in a variety of biological processes, such as morphogenesis during the embryonic stage, cell selection during lymphoid development, tissue repair after injury, regression of inflammation, elimination of cells at risk of developing into a tumor and lymphocyte-mediated killing [19]. A balance of proliferation and apoptosis is essential for the tissue homeostasis [20,21].

Apoptosis as mechanism of controlled cell death is a well-controlled process and progresses through a series of morphological and biochemical phases, including chromatin condensation and activation of proteolytic enzymes [22,23]. A number of mediators involved in the induction of apoptosis have been identified during recent years. Probably the most thoroughly characterized death receptor is the cell membrane receptor Fas (CD 95), a member of the TNF receptor family. Cross-linking of Fas, either via specific antibodies or via its specific ligand, activates a cascade reaction of caspases, which are responsible for induction of membrane alterations, breakdown of cellular constituents and DNA, and finally cell death [19].

In the kidney beside the damage induced by infiltrating inflammatory cells the relationship among resident cell proliferation and apoptosis in glomeruli determines the outcome in various glomerulonephritides [13]. Several groups reported that apoptosis plays an important role in the repair process in experimental and human glomerulonephritis [24,25]. Apoptosis has an additional role in the sclerosing process in the glomeruli [26]. Cell number abnormalities are frequent in renal diseases, and range from the hypercellularity of postinfectious glomerulonephritis to the cell depletion of chronic renal atrophy. Death ligands and receptors, such as TNF and Fas-ligand, pro-apoptotic and anti-apoptotic Bcl-2 family members and caspases have all been shown to participate in apoptosis regulation in the course of renal injury [27]. Some reports suggest that altered apoptotic signaling and regulatory mechanisms contribute to further progressive renal impairment, tubular atrophy, interstitial fibrosis, and glomerulosclerosis in a model of focal and segmental glomerulosclerosis in rats [28].

In the glomerulus a balance between endothelial, mesangial and visceral epithelial cells and their extracellular matrix is essential for structural and functional integrity. During glomerular injury function and morphology of these cells are altered. Intrinsic cell proliferation in the glomerulus is regulated by a large number of mediators and growth factors like IL-10 [29], insulin-like growth factors [30] and platelet derived growth factor [31]. Some of these factors also influence apoptosis in the glomerulus [32].

The basis for the experiments performed in this work was the hypothesis that chemokines and chemokine receptors expressed by intrinsic renal cells may be involved both in the maintenance of glomerular homeostasis in normal adult human kidney and regulation of glomerular cell numbers during disease states. We previously showed constitutive expression of CCR7 protein and its ligand secondary lymphoid tissue chemokine (SLC/CCL21) in human renal tissue. In immunohistochemistry we found a clear staining pattern for SLC/CCL21 on podocytes and CCR7 on MC during nephrogenesis and in adult kidney. Also constitutive mRNA expression has been shown for CCR7 in cultured MC and for SLC/CCL21 in isolated human glomeruli. Furthermore it was demonstrated that mesangial CCR7 is functionally active since for example SCL/CCL21 induced a dose-dependent migration of MC [12]. We therefore investigated the influence of chemokines on MC growth and found an significant increase in proliferation of MC after stimulation with SLC/CCL21 in a dose-dependent manner. To study the role of SLC/CCL21 in MC apoptosis cell death was induced by activating mesangial Fas/CD95 receptors. SLC/CCL21 was found to prevent MC apoptosis as shown by cell cycle analysis and Hoechst stain. Since caspase-3 assays revealed impaired activity it can be assumed that this molecule is involved in chemokine-influenced intracellular apoptosis pathways in MC. The finding of an anti-apoptotic function of SLC/CCL21 is novel. At present SLC/CCL21 is known to be constitutively produced by high endothelial venules and stromal cells within T cell zones of lymph nodes [33]. Its corresponding receptor CCR7 is expressed on naive T cell subpopulations and up-regulated by maturing dendritic cells [34]. Therefore this chemokine/chemokine receptor pair was described as an prototypic model for the homing of immune cells to lymphoid tissue [35,36]. Anti-apoptotic effects of chemokines seem not to be restricted to SLC/CCL21. The CX3CR1-binding chemokine fractalkine which is constitutively expressed on neuronal cells has been demonstrated as survival factor for brain microglia in Fas-induced cell death [37].

A role of the chemokine receptor CXCR3 in inflammatory glomerular disease has been proposed before by the group of Romagnani et al. since a mesangial expression of CXCR3 in biopsies from patients with mesangioproliferative glomerulonephritis could be demonstrated [11]. We therefore investigated the effects of the CXCR3 ligands IP-10/CXCL10 and Mig/CXCL9 on MC and also found a concentration dependent increase of proliferation of MC after stimulation with these chemokines. Interestingly, in contrast to the effect observed with SLC/CCL21 both IP-10/CXCL10 and Mig/CXCL9 had no effect on Fas induced apoptosis of MC.

The third chemokine receptor of interest was CCR1 since our group has demonstrated functionally active expression of CCR1 on human MC, inducible after stimulation with a combination of the proinflammatory cytokines TNF-α, IL-1β and IFN-γ [9]. Futhermore upregulation of CCR1 expression is also known in an animal model for immune complex glomerulonephritis [38]. In contrast to the effects observed with CCR7 and CXCR3 ligands, stimulation of MC with the CCR1 ligand RANTES/CCL5 had no effect on cell proliferation and apoptosis. In this context an article of Topham et al. is of special interest. This group demonstrated that CCR1 may have anti-inflammatory functions since mice negative for CCR1 showed enhanced Th1 immune responses and worsened histology in a model of nephrotoxic serum nephritis [39]. Our group showed in a model of horse apoferritin (HAF)-induced glomerulonephritis that CC chemokine ligand 5/RANTES chemokine antagonists aggravate glomerulonephrtis despite reduction of glomerular leukocyte infiltration. These findings were associated with an enhancing effect of the CCL5/RANTES analogs on the macrophage activation state *in vitro *and *in vivo*. The humoral response and the Th1/Th2 balance in HAF-glomerulonephritis and mesangial cell proliferation *in vitro *were not affected by the CCL5/RANTES analogs [40]. Therefore also the effects of the CCR1 blocker Met-RANTES were studied but showed no influence on MC proliferation and apoptosis.

## Conclusions

In summary it is tempting to speculate that the different effects of SLC/CCL21 and IP-10/CXCL10 and Mig/CXCL9 on proliferation and apoptosis of MC represent specialized functions of chemokine receptos on non-immune cells. CCR7 could be a chemokine receptor important for the development of the glomerular architecture during ontogenesis and for maintaining glomerular homeostasis in adult human kidney. CXCR3 may have its main functions important for mesangial expansion in mesangioproliferative disease. In contrast, the chemokine receptor CCR1 and its ligands RANTES/CCL5 and Met-RANTES seems not to have a special impact for the regulation of proliferation and apoptosis on MC. CCR1 may be important for local immunomodulation especially in glomerular inflammation. The chemokines and their receptors we have analyzed seem to be part of a complex system of factors which regulate proliferation and apoptosis in kidney and therefore play a pivotal role in regulation of glomerulogenesis, during glomerular injury and in homeostatic balance in the glomerulum. Studying these locally synthesized chemokines and their interaction with corresponding receptors on non-immune cells deserves further investigation and will reveal novel chemokine/chemokine receptor functions far beyond their orignal functions in guiding inflammatory cells to sites of tissue injury.

## Competing interests

None declared.

## Authors' contributions

All authors were involved in experimental procedures and manuscript preparation.

## Abbreviations

mesangial cell (MC); chemokines monocyte chemoattractant protein-1 (MCP-1); regulated upon activation, normal T cell expressed and secreted (RANTES/CCL5); interferon-γ (IFN-γ)-inducible protein of 10 kD (IP-10/CXCL10); monokine induced by IFN-γ (Mig/CXCL9); (3-(4,5-dimethylthiazol-2-yl)-2,5-diphenyl-tetrazolium bromide (MTT)

## Pre-publication history

The pre-publication history for this paper can be accessed here:



## References

[B1] Luster AD (1998). Chemokines: Chemotactic cytokines that mediate inflammation. N Engl J Med.

[B2] Muller A, Homey B, Soto N, Ge N, Catron D, Buchanan ME, McClanahan T, Murphy E, Yuan W, Wagner SN, Barrera JL, Mohar A, Verastegui E, Zlotnik A (2001). Involvement of chemokine receptors in breast cancer metastasis. Nature.

[B3] Tachibana K, Hirota S, Iizasa H, Yoshida H, Kawabata K, Kataoka Y, Kitamura K, Matsushima N, Yoshida N, Nishikawa S, Kishimoto T, Nagasawa T (1998). The chemokine receptor CXCR4 is essential for vascularization of the gastrointestinal tract. Nature.

[B4] Zou YR, Kottmann AH, Kuroda M, Taniuchi I, Littmann DR (1998). Function of the chemokine receptor CXCR4 in haematopoiesis and in cerebellar development. Nature.

[B5] Mackay CR, Chemokines (1997). What chemokine is that?. Curr Biol.

[B6] Rollins BJ (1997). Chemokines. Blood.

[B7] Murphy PM, Baggiolini M, Charo IF, Herbert CA, Horuk R, Matsushima K, Miller LH, Oppenheim JJ, Power CA (2000). International union of pharmacology. XXII. Nomenclature for chemokine receptors. Pharmacol Rev.

[B8] Segerer S, Nelson PJ, Schlöndorff D (2000). Chemokines, chemokine receptors, and renal disease: from basic science to pathophysiologic and therapeutic studies. J Am Soc Nephrol.

[B9] Banas B, Luckow B, Möller M, Klier C, Nelson PJ, Schadde E, Brigl M, Halevy D, Holthöfer H, Reinhart B, Schlöndorff D (1999). Chemokine and chemokine receptor expression in a novel human mesangial cell line. J Am Soc Nephrol.

[B10] Gomez-Chiarri M, Hamilton TA, Egido J, Emancipator SN (1993). Expression of IP-10, a lipopolysaccharide- and interferon-gamma-inducible protein, in murine mesangial cells in culture. Am J Pathol.

[B11] Romagnani P, Beltrame C, Annunziato F, Lasagni L, Luconi M, Galli G, Cosmi L, Maggi E, Salvadori M, Pupilli C, Serio M (1999). Role for interactions between IP-10/Mig and CXCR3 in proliferative glomerulonephritis. J Am Soc Nephrol.

[B12] Banas B, Wörnle M, Berger T, Nelson PJ, Cohen CD, Kretzler M, Pfirstinger J, Mack M, Lipp M, Gröne HJ, Schlöndorff D (2002). Roles of SLC/CCL21 and CCR7 in human kidney for mesangial proliferation, migration, apoptosis and tissue homeostasis. J Immunol.

[B13] Harrison DJ (1988). Cell death in the diseased glomerulus. Histopathology.

[B14] Schlöndorff D, Nelson PJ, Luckow B, Banas B (1997). Chemokines and renal disease. Kidney Int.

[B15] Heeg K, Reimann J, Kabelitz D, Hardt C, Wagner H (1985). A rapid colorimetric assay for the determination of IL-2-producing helper T cell frequencies. J Immunol Methods.

[B16] Gonzales-Cuadrado S, Lopez-Armada MJ, Gomez-Guerrero C, Subira D, Garcia-Sahuquillo A, Ortiz-Gonzales A, Neilson EG, Egido J, Ortiz A (1996). Anti-Fas antibodies induce cytolysis and apoptosis in cultured human mesangial cells. Kidney Int.

[B17] Berger T, Brigl M, Hermann JM, Vielhauer V, Luckow B, Schlöndorff D, Kretzler M (2000). The apoptosis mediator mDAP-3 is a novel member of a conserved family of mitochondrial proteins. J Cell Sci.

[B18] Fernandes-Alnemri T, Litwack G, Alnemeri ES (1994). CPP32, a novel human apoptotic protein with homology to Caenorhabditis elegans cell death protein Ced-3 and mammalian interleukin-1 beta-converting enzyme. J Biol Chem.

[B19] Roos A, Sato T, Maier H, van Kooten C, Daha MR (2001). Induction of renal cell apoptosis by antibodies and complement. Exp Nephrol.

[B20] Krammer PH (2000). CD95's deadly mission in the immune system. Nature.

[B21] Ortiz A, Lorz C, Egido J (1999). New kids in the block: the role of FasL and Fas in kidney damage. J Nephrol.

[B22] Kerr JF, Wyllie AH, Currie AR (1972). Apoptosis: a basic biological phenomen with wide-ranging implications in tissue kinetics. Br J Cancer.

[B23] Hale AJ, Smith CA, Sutherland LC, Stoneman VE, Longthorne VL, Culhane AC, Wiliams GT (1996). Apoptosis: molecular regulation of cell death. Eur J Biochem.

[B24] Baker AJ, Mooney A, Hughes J, Lombardi D, Jonson RJ, Savill J (1994). Mesangial cell apoptosis: The major mechanism for resolution of glomerular hypercellularity in experimental mesangial proliferative nephritis. J Clin Invest.

[B25] Shimizu A, Kitamura H, Masuda Y, Ishizaki M, Sugisaki Y, Yamanaka N (1995). Apoptosis in the repair of experimental proliverative glomerulonephritis. Kidney Int.

[B26] Shimizu A, Masuda Y, Kitamura H, Ishizaki M, Sugisaki Y, Yamanaka N (1996). Apoptosis in progressive crescent glomerulonephritis. Lab Invest.

[B27] Ortiz A, Lorz C, Justo P, Catalan MP, Egido J (2001). Contribution of apoptotic cell death to renal injury. J Cell Mol Med.

[B28] Wang W, Tzanidis A, Divjak M, Thomson NM, Stein-Oakley AN (2001). Altered signaling and regulatory mechanisms of apoptosis in focal and segmental glomerulosclerosis. J Am Soc Nephrol.

[B29] Kitching AR, Katerelos M, Mudge SJ, Tipping PG, Power DA, Holdsworth SR (2002). Interleukin-10 inhibits experimental mesangial proliferative glomerulonephritis. Clin Exp Immunol.

[B30] Feld SM, Hirschberg R, Artishevsky A, Nast C, Adler SG (1995). Insulin-like growth factor I induces mesangial proliferation and increases mRNA and secretion of collagen. Kidney Int.

[B31] Floege J, Eng E, Young BA, Alpers CE, Barrett TB, Bowen-Pope DF, Johnson RJ (1993). Infusion of platelet-derived growth factor or basic fibroblast growth factor induces selective glomerular mesangial cell proliferation and matrix accumulation in rats. J Clin Invest.

[B32] Sano H, Ueda Y, Takakura N, Takemura G, Doi T, Kataoka H, Murayama T, Xu Y, Sudo T, Nishikawa S, Nishikawa S, Fujiwara H, Kita T, Yokode M (2002). Blockade of platelet-derived growth factor receptor-beta pathway induces apoptosis of vascular endothelial cells and disrupts glomerular capillary formation in neonatal mice. Am J Pathol.

[B33] Campbell JJ, Bowman EP, Murphy K, Youngman KR, Siani MA, Thompson DA, Wu L, Zlotnik A, Butcher EC (1998). 6-C-kine (SLC), a lymphocyte adhesion triggering chemokine expressed by high endothelium, is an agonist for the MIP-3β receptor CCR7. J Cell Biol.

[B34] Dieu MC, Vanbervliet B, Vicari A, Bridon JM, Oldham E, Ait-Yahia S, Briere F, Zlotnik A, Lebecque S, Caux C (1998). Selective recruitment of immature and mature dendritic cells by distinct chemokines expressed in different anatomic sites. J Exp Med.

[B35] Cyster JG (2000). Leukocyte migration: scent of the T zone. Curr Biol.

[B36] Sallusto F, Lenig D, Forster R, Lipp M, Lanzavecchia A (1999). Two subsets of memory T lymphocytes with distinct homing potentials and effector functions. Nature.

[B37] Boehme SA, Lio FM, Maciejewski-Lenoir D, Bacon KB, Conlon PJ (2000). The chemokine fractalkine inhibits Fas-mediated cell death of brain microglia. J Immunol.

[B38] Anders HJ, Vielhauer V, Kretzler M, Cohen CD, Segerer S, Luckow B, Weller L, Gröne HJ, Schlöndorff D (2001). Chemokine and chemokine receptor expression during initiation and resolution of immune complex glomerulonephritis. J Am Soc Nephrol.

[B39] Topham PS, Csizmadia V, Soler D, Hines D, Gerard CJ, Salant DJ, Hancock WW (1999). Lack of chemokine receptor CCR1 enhances Th1 responses and glomerular injury during nephrotoxic nephritis. J Clin Invest.

[B40] Anders HJ, Frink M, Linde Y, Banas B, Wörnle M, Cohen CD, Vielhauer V, Nelson PJ, Grone HJ, Schlöndorff D (2003). CC chemokine ligand 5/RANTES chemokine antagonists aggravate glomerulonephritis despite reduction of glomerular leukocyte infiltration. J Immunol.

